# Diagnosing Eosinophilic Colitis: Histopathological Pattern or Nosological Entity?

**DOI:** 10.6064/2012/682576

**Published:** 2013-05-09

**Authors:** Alan W. H. Bates

**Affiliations:** Research Department of Pathology, University College London, London WC1E 6BT, UK

## Abstract

Reports of  “eosinophilic colitis”—raised colonic mucosal eosinophil density in patients with lower gastrointestinal symptoms—have increased markedly over the last fifteen years, though it remains a rarity. There is no consensus over its diagnosis and management, and uncertainty is compounded by the use of the same term to describe an idiopathic increase in colonic eosinophils and an eosinophilic inflammatory reaction to known aetiological agents such as parasites or drugs. In patients with histologically proven colonic eosinophilia, it is important to seek out underlying causes and careful clinicopathological correlation is advised. Because of the variability of eosinophil density in the normal colon, it is recommended that histological reports of colonic eosinophilia include a quantitative morphometric assessment of eosinophil density, preferably across several sites. Few reported cases of “eosinophilic colitis” meet these criteria. As no correlation has been shown between colonic eosinophil density and symptoms in older children or adults, it is suggested that treatment should be directed towards alleviation of symptoms and response to treatment assessed clinically rather than by histological estimates of intramucosal eosinophils.

## 1. Introduction

Eosinophils are predominantly tissue-dwelling cells; at any time comparatively few are circulating in the blood. Most are to be found in the bone marrow, where they are formed, and in the lamina propria of the gastrointestinal tract, of which they are a normal component, acting as a protective mechanism against parasites. Eosinophils respond to stimuli, including trauma, infection, and allergens, by degranulating to release inflammatory mediators including leukotrienes, vasoactive intestinal polypeptide, tumour necrosis factor, and interleukins [1]. Eosinophil density in the colon is increased in various disorders including food allergy, parasitic infections, and inflammatory bowel disease, but in some patients no underlying gastrointestinal pathology is identified and in these cases a diagnosis of primary eosinophilic colitis is sometimes made.

This rare primary form of eosinophilic colitis has been the subject of fewer than a hundred case reports, including cases from several small series, and its diagnosis and treatment have been discussed in some recent reviews of eosinophilic gastrointestinal disease [1, 2]. Given the nonspecific symptoms—most commonly abdominal pain, constipation, diarrhea, and rectal bleeding—that are associated with eosinophilic colitis, the lack of distinctive clinical findings, and its relapsing-remitting course, it is necessary to establish the diagnosis by examination of colonic biopsy material. Eosinophils are easily visible in routine haematoxylin- and-eosin-stained paraffin-embedded sections and can be assessed semiquantitatively. At present, no consensus has been reached on the histological criteria required to make the diagnosis, and this makes comparisons between reported cases difficult. This paper reviews the histopathological findings in previously reported cases of eosinophilic colitis and considers whether they represent a nonspecific histological reaction pattern or a distinct pathological entity. On the basis of a literature survey, published data on normal eosinophil density, and the author's experience of histological examination of material with prominent eosinophilic infiltrates at a tertiary referral centre, some recommendations for diagnosis and management will be made.

## 2. Historical Background

Eosinophilic colitis was first described in 1936 and the term first appeared in the literature in English in 1959 [3, 4]. Subsequently, the term “eosinophilic colitis” was used to describe appearances in parasitic infestation of the colon and in milk-intolerant neonates [5, 6]. In 1985 Naylor and Pollet reviewed twenty-two cases of eosinophilic colitis: no common aetiology was identified, though food allergies, drug reactions, and worms were reported in a minority of patients [7]. In Naylor and Pollet's review and in a subsequent paper by Moore and others, eosinophilic colitis was regarded as a subset of eosinophilic gastroenteritis characterized by an eosinophilic infiltrate in the colon in association with peripheral eosinophilia and symptoms including abdominal pain, nausea, vomiting, and diarrhoea [8].

The publication of a series of thirteen cases of allergic colitis that had initially presented before two years of age identified a specific subtype of eosinophilic colitis: a treatable disorder of early childhood that was caused by food allergy (usually to eggs, milk, or soya) and was of limited duration, typically remitting entirely with an appropriate exclusion diet [9]. Two years later, a rare tumoural form of eosinophilic colitis, which can present as a colonic mass, was named by Minciu and others, who identified six cases in a review of the literature [10].

## 3. Incidence

A recent review described eosinophilic colitis as “exceptionally rare” [11]. This is true in terms of the paucity of well attested cases in the literature, but it has been suggested that it may be more common than is generally supposed: in 2004 a continuous series of colonoscopies performed at Great Ormond Street Hospital over a period of one year for any indication included twelve cases of eosinophilic colitis in children over the age of one year and five cases in infants: eosinophil density was not specified [12]. Over a seven-year period at the author's tertiary referral centre a mean colonic eosinophil density of >20/high-power field (HPF) was reported in twelve children over the age of one year. This represents just over 1% of colonoscopic biopsy series examined, though in some cases this degree of eosinophilia may be a normal variation rather than a pathological process [13]. The principal difficultly in interpreting such findings is the shortage of quantitative data regarding the normal colonic eosinophil population.

## 4. Normal Eosinophil Numbers

Given the wide age range in reported cases of eosinophilic colitis, ethical considerations make it unlikely that tissue from normal controls will be obtained from an age-matched population. In practise, most “normal” colonic samples are gathered from symptomatic patients in whom no underlying pathology can be detected. While this source of controls is methodologically suboptimal, it does have the practical advantage that for histopathologists the requirement is usually to distinguish eosinophilic colitis from other pathologies in symptomatic patients. 

Consent for sampling the colon at autopsy for the purposes of research can also be difficult to obtain, so the work of Lowichik and Weinberg on colonic eosinophil density in biopsies taken from paediatric autopsies provides especially valuable data. Colonic samples from 39 children showed a mean of 17 eosinophils per high-power field but the range was wide (1–52/HPF) and 28% averaged more than 20/HPF. All the samples came from children who had died from trauma and had no known history of gastrointestinal disease. The study also demonstrated a gradient of eosinophil density from proximal to distal, from a mean of 35/HPF in the caecum to 10/HPF in the rectum [14]. A more detailed study on endoscopic material was carried out by DeBrosse and others, who also found a gradient of eosinophil density from ascending colon to rectum (20/HPF and 8/HPF, resp.) and a wide range (up to 50/HPF), comparable to the findings of Lowichik and Weinberg [15]. Since the cases studied by DeBrosse and colleagues were selected from a group initially reported as histologically normal, they may, if anything, underestimate eosinophil numbers in typical endoscopic series. The proximal to distal gradient of eosinophil distribution has also been described by Gonsalves [16]. Geographic variation in eosinophil density in the normal colon has also been reported and there may also be variations in response to seasonal antigenic changes and subclinical infections [17, 18]. 

## 5. Diagnostic Criteria

The simplest definition of eosinophilic colitis is one of symptomatic raised colonic eosinophil density. Although transmural eosinophilia has been described in resection specimens, assessment of colonic eosinophils is usually made on mucosal biopsies and to standardize the diagnosis of eosinophilic colitis it will be necessary to set a limit above which the diagnosis is made. As eosinophil density varies with site, one could set a limit for each site or use a mean value across the whole colon. Hitherto, neither method has been applied consistently. For practical reasons eosinophil density is usually estimated semiquantitatively, by counting the number of eosinophils in three or more high-power microscopic fields and calculating the mean. Drawbacks with this approach are variability in field size according to the equipment used (it is important if employing this method to state the field size in mm^2^), selection of fields (eosinophils may be more numerous near lymphoid follicles), and criteria for including cells in the count. Eosinophils are readily identified in haematoxylin- and-eosin-stained sections but some studies include all eosinophils seen and others only those where the nucleus is visible. 

The difficulty in reaching a consistent assessment of eosinophil density is further complicated by the lack of an accepted figure above which eosinophil density is to be regarded as abnormal [19]. A density of greater than 60 eosinophils/10 HPF has been suggested [20, 21] though this is below the mean levels found at autopsy in children without known colonic disease [14]. Others have adopted 30/HPF [22], a figure which, though corresponding with a histologically conspicuous eosinophilic infiltrate, would be expected to include some normal cases: Lowichick and Weinberg found a mean of 17 eosinophils/HPF (89/mm^2^) and observed up to 52/HPF in individuals with no known gastrointestinal disease, and DeBrosse and others found up to 50 eosinophils/HPF in apparently normal colons (Table 1). At present, therefore, there are no accepted criteria for distinguishing colonic eosinophil density at the upper range of normal from a pathological increase diagnostic of primary eosinophilic colitis.

**Figure 1:**
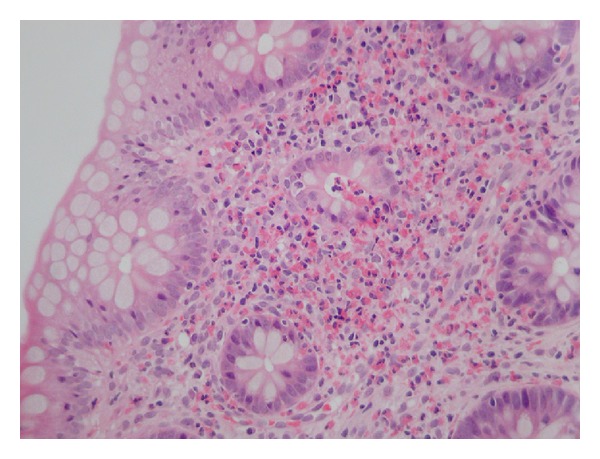
A heavy colonic eosinophilic infiltrate of >100 eosinophils/HPF with eosinophilic cryptitis and microabscess formation.

In addition to counts of total eosinophil density, some studies have assessed degranulation as an indicator of eosinophil activation. It is possible to observe degranulation in routinely stained sections and to grade it semiquantitatively; however, it is not known if the trauma of biopsy could provoke degranulation of otherwise inactive eosinophils. Degranulation appears to correlate with eosinophil density, though not with symptoms [13]. Theoretically, eosinophil degranulation may lead to lysosomal, oxidative, and cytotoxic damage and, in the long term, localized fibrosis [2], though the latter has not been reported in the gastrointestinal tract. In contrast to eosinophilic oesophagitis, where there are macroscopically visible changes including linear furrows and exudates, eosinophilic colitis is not associated with characteristic endoscopic or histological architectural changes. Other histological findings that might be seen in patients with raised colonic eosinophil density are eosinophil microabscesses, eosinophilic cryptitis, and eosinophils within the surface epithelial compartment (Figure 1), though in our experience the latter can be a normal finding. 

In most suspected cases of eosinophilic colitis only mucosal biopsies are available for assessment. It may be, however, that eosinophilic infiltration of deeper layers of the colon contributes to symptoms. In a study of small bowel resections, eosinophilic enteritis was divided into mucosa-predominant, muscularis propria predominant, and serosa-predominant [23]. There are no such studies of colonic resections, but eosinophilic ganglionitis in Meissner's plexus has been reported in three cases of pseudoobstruction of the colon in children, leading to the suggestion that the symptoms of eosinophilic infiltration of the colon may be due to ganglionitis-induced dysmotility [24]. 

## 6. Differential Diagnosis

In recent reviews, Alfadda and others suggested making the diagnosis of eosinophilic colitis contingent on the presence of a dense eosinophilic infiltrate in one or more segments of the colon, without evidence of parasites or other underlying disease [2, 11]. Before a diagnosis of primary or idiopathic eosinophilic colitis can be made, many other potential causes of an eosinophilic response need to be excluded.

Colonic eosinophilia has been described in association with pinworms, roundworms, and whipworms [25–27] and in dientamoebiasis [28]. Helminth larvae may not always be seen in histological sections and so it is important for the pathologist to be aware of the endoscopic findings and any relevant history of travel. If parasitic aetiology is suspected, stool examination or specific serology may be performed.

Drugs reported to cause colonic eosinophilia include nonsteroidal anti-inflammatories [29], tacrolimus [30], carbamazrpine [31], rifampicin [32], sulphasalazine [33], and naproxen [29]. Because of the rarity of eosinophilic colitis, a causal link with specific drug therapy can be difficult to establish; particular attention should be paid to the temporal relationship between drug administration and symptoms, and colonic eosinophilia should not be attributed to a drug reaction without adequate clinicopathological correlation [18].

Eosinophilic colitis has been linked with scleroderma [34] but not systemic lupus erythematosus, though the latter is associated with eosinophilic enteritis [67]. Eosinophilic colitis is also well described after liver transplantation in children [45]. Recently, two cases of “eosinophilic colitis” in association with childhood autism have been reported though eosinophil density was not quantified [68]. 

Since tissue eosinophils are increased in many chronic inflammatory conditions, there is a potential for misdiagnosis of early or inactive inflammatory bowel disease as eosinophilic colitis [69]. In contrast to quiescent inflammatory bowel disease, the architecture of the colonic crypts in eosinophilic colitis is normally preserved. In doubtful cases with prominent eosinophils, especially if symptoms worsen, rebiopsy after several months may be necessary to exclude inflammatory bowel disease. One study has described Crohn's type colitis with a heavy eosinophilic infiltrate as eosinophilic-Crohn overlap colitis [70]. Pensabene and others found a higher overall colonic eosinophil density in children with inflammatory bowel disease compared to those with food allergies [71], which suggests that it may not be possible reliably to diagnose eosinophilic colitis in the presence of inflammatory bowel disease, especially Crohn's disease, where eosinophils are typically more numerous than in ulcerative colitis [72]. Patients with eosinophilic colitis occasionally show peripheral eosinophilia, and there is a statistically significant association between colonic eosinophil density and elevated total serum IgE levels [13]. It has been proposed that gut eosinophilic disorders are IgE-mediated through the high-affinity receptor FcepsilonRI [73].

## 7. Association with Other Eosinophilic or Allergic Conditions

Eosinophilic colitis has often been discussed in association with eosinophilia elsewhere in the gut, and terms such as “eosinophilic gastrointestinal disorder” (EGID) have been employed to refer to any hypereosinophilic condition involving part of the oesophago-gastrointestinal tract [74]. However, evidence linking eosinophilic colitis with eosinophilic infiltration elsewhere in the gut is lacking: a recent review of EGID concluded that eosinophilic colitis has a different pathophysiology and is probably best regarded as a separate entity [75]. 

The most common eosinophilic disorder of the gut, eosinophilic oesophagitis, appears not to be associated with eosinophilia in the colon. No case of coincident eosinophilic oesophagitis and eosinophilic colitis has been reported: in my own institution, where there have been six reports of eosinophilic colitis and 133 of eosinophilic oesophagitis in the past ten years, no patient had both findings. Furthermore, while there has been a significant rise in the incidence of eosinophilic oesophagitis in the past two decades, eosinophilic colitis remains rare [76]. 

Since the small bowel is infrequently biopsied, data on eosinophilic enteritis are relatively sparse; one study did show an association between a high eosinophil density in the colon and terminal ileal eosinophilia, but this could represent a phenomenon akin to backwash ileitis in ulcerative colitis [13]. Interestingly, just over half of patients with a diagnosis of eosinophilic colitis who contributed to a worldwide web-based registry self-reported another type of EGID [77]. Though histopathological correlation of this claim is not available, this potential link between eosinophilic colitis and enteritis merits further investigation.

On the basis of similarities in pathogenesis, a potential association between bronchial asthma and eosinophilic gastroenteritis has been mooted [78], but in practice eosinophilic colitis has not been linked to a history of atopy. DeBrosse and others looked at seventeen patients with a history of atopy (asthma, allergic rhinitis, or eczema) and found no significant difference in colonic eosinophil numbers compared with nonatopic children [15].

The bimodal distribution of the incidence of eosinophilic colitis by age, with peaks for neonates and young adults [75], raises the question of whether the infantile form of the condition is nosologically distinct. Some authors have classified infantile colonic eosinophilia as a distinct entity [79] under a variety of names, including allergic eosinophilic proctocolitis and food protein-induced enterocolitis, most using the latter term or a close variant of it [80]. Allergic eosinophilic proctocolitis is an inflammatory reaction to a foreign protein (either consumed directly or from the maternal diet through breast milk), which occurs in unweaned babies, generally under the age of three months and remits after exclusion of the protein [81, 82]. In a few cases, food protein-induced enterocolitis or proctocolitis has been reported to lead to dehydration and shock [83]; more often, however, symptoms are limited to mild diarrhoea and low-grade rectal bleeding [84]. Allergy to cow milk is a common aetiology in formula-fed infants, but dietary proteins from soya, eggs, or pulses may be the culprits in breast-fed infants, in whom food protein-induced enterocolitis is most often a reaction to transferred maternal proteins [21]. After weaning, infants who continue to ingest the allergen may go on to develop food-specific IgE sensitivity [85]. Prognosis is very good after the trigger food is identified [86].

## 8. Management

After dietary restriction in allergic eosinophilic proctocolitis the number of eosinophils falls to within the normal range within twelve months [87]. Elimination, oligoantigenic or elemental diets also provide symptomatic relief and in time the offending food may be reintroduced as most patients acquire tolerance before five years of age [88]. In such cases normalization of eosinophil density can provide conformation that the condition has resolved, but since eosinophil numbers in the colon are variable (at least in older children) it seems preferable to treat symptoms rather than histopathological appearances. A recent case series of older symptomatic children in whom colonic eosinophilia was the principal pathological diagnosis showed no association between eosinophil density and symptoms, history of atopy, inflammatory markers, or clinical outcome. Furthermore, follow-up demonstrated no correlation between changes in the tissue eosinophil count and improvement of symptoms [13].

In older children and adults, a spectrum of treatments other than dietary restriction have been employed. These include glucocorticoids, azathioprine, montelukast, and ketotifen [2]. There is no consensus on treatment of eosinophilic colitis after infancy as the condition seems to follow a relapsing-remitting course and may, as in the infantile form and some cases of eosinophilic oesophagitis, be self-limiting. Treatment should be targeted at symptom reduction since tissue eosinophilia appears to be a poor indicator of disease severity. 

## 9. Is Eosinophilic Colitis a Nosological Entity?

**Figure 2:**
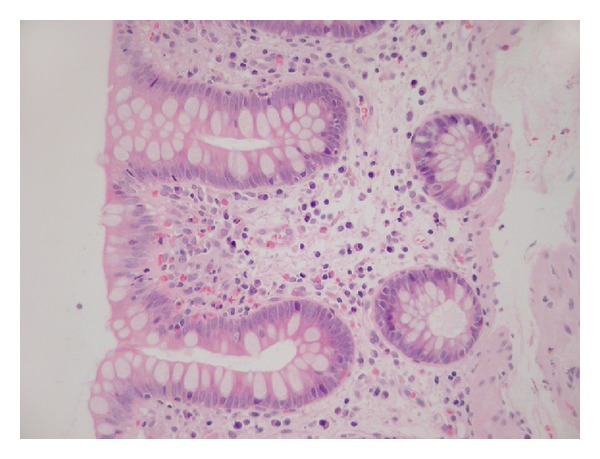
Proximal colon showing 20–25 eosinophils/HPF within the lamina propria. This is not a pathological finding.

Having eliminated secondary causes of colonic eosinophilia and the infantile food allergic disorders sometimes classified separately, is there a residual primary form of eosinophilic colitis? The increasing number of case reports of eosinophilic colitis in the literature in English appears to lend support to the emergence of a new entity, but in the majority of these cases there was no clearly defined tissue diagnosis (Table 2). Of thirty-four papers in English reporting “eosinophilic colitis” since 1959, eosinophil density was quantified in only seven (Figure 2). In these, the density considered to indicate the diagnosis ranged from >20/HPF anywhere in the colon [35] to >120/HPF [52]. The former figure lies with the normal range, and in our experience this density of eosinophils is not uncommon in the proximal colon. There remain only seven cases in total of primary eosinophilic colitis in the literature (four adults and three children) in which measured eosinophil density was greater than 30/HPF [22, 37, 41, 44]. Of these, one was drug related and one was associated with caecal volvulus; the remaining five (two adults and three children) were idiopathic. Other published reports of “eosinophilic colitis” have included histological examination of colonic biopsies but without quantification of eosinophil density. In some of these cases a “massive” or “heavy” eosinophilic infiltrate was described while in others it was “patchy” with “varying” numbers of eosinophils [46, 51, 55, 56]. 

Colonic eosinophilia may be a significant finding in some symptomatic individuals but there has been no consistency regarding the cut-off point above which eosinophil density should be regarded as increased, the region of colon to be assessed, or the number of microscopic fields examined. In view of the paucity of quantitative data, the large number of conditions and drugs that have been associated with secondary eosinophilia, the variability of symptoms, and the lack of correlation between symptoms and eosinophil density, eosinophilic colitis is best regarded as a nonspecific reaction pattern rather than a nosological entity. 

## 10. Conclusion

Colonic eosinophilia in adults and children over one year of age may represent a nonspecific reaction to antigen exposure or underlying disease. The uncertainty over diagnosis emphasises the need for proper clinicopathological correlation. In my own institution, all paediatric endoscopic series are reviewed at clinicopathological meetings attended by pathologists and clinicians. Mean eosinophil counts per high-power field are given in the histopathology report in cases where they form a conspicuous part of the infiltrate, but the term “eosinophilic colitis” is not used.

While experience shows that a small proportion of children and adults investigated for lower gastrointestinal symptoms have a conspicuous eosinophilic infiltrate within the lamina propria of the colon, in practice, eosinophil density is rarely greater than the upper range that has been observed in supposedly normal series, nor has any study shown correlation between colonic eosinophil density and symptoms, outcome, or other pathology.

Necessarily, all patients biopsied will be symptomatic, and there may well be a clinical case to be made for treatment. While reduction in eosinophil density in subsequent biopsies might be expected to indicate response to treatment, it is important to remember that this has yet to be demonstrated, and the aim of treatment should be to alleviate symptoms rather than to normalize the patient's histology.

## Figures and Tables

**Table 1:** Eosinophil densities in normal colon and eosinophilic colitis.

Normal colonic eosinophil density	
Authors	Overall range (mean) of eosinophils/HPF
Lowichik and Weinberg 1996 [14]	1–52 (17)
DeBrosse et al. 2006 [15]	1–50 (15)

Suggested minimum eosinophil density for diagnosis of eosinophilic colitis	
Authors	Eosinophils/HPF

Odze et al. 1993 [20]	6
Hwang et al. 2007 [21]	6
Lee and Kim 2010 [22]	30

**Table 2:** Eosinophilic colitis in the literature.

Author(s)	Age in years (y) or months (m)	Sex	Eosinophil numbers
Melton et al. [35]	2 m, 4 m, 3 y, 53 y, 73 y	all M	>20/HPF
Yamada et al. [36]	8 m, 2 m, 3 m, 8 m, neonate	3 M, 2 F	No quantification
Basilious and Liem [37]	9 y, 9 y, 5 y	2 M, 1 F	Up to 60/HPF
Lee and Kim [22]	50 y	F	>30/HPF
Durilova et al. [38]	1–12 m	13 M, 7 F	No quantification; no biopsies in some cases
Piscaglia et al. [39]	46 y	M	No quantification
Chen et al. [40]	53 y	M	No quantification
Shim et al. [41]	83 y	F	>100/HPF
Sussman et al. [42]	52 y	M	“Multiple”
Ertuğrul et al. [43]	46 y	F	“Clusters”
Velchuru et al. [44]	48 y	F	80/HPF (colectomy)
Lee et al. [45]	11 m, 6 m, 5 m, 2 y, 7 m, 7 m, 7 m, 8 m, 10 m, 18 m, 9 m, 1 y, 7 m, 11 m	4 M, 9 F	>15/HPF in rectum
Ohtsuka et al. [46]	Infants	1 M, 1 F	“Massive”
Lewis et al. [47]	78 y	F	“Numerous”
Inamura et al. [48]	35 y	M	“Numerous”
Kandaswamy et al. [49]	65 y	M	No quantification
Saeed et al. [30]	3 y, 4 y, 8 y	all M	“Raised”
Sierra Salinas et al. [50]	Infants	8 M, 5 F	“Increased” only 9 of 13 were biopsied
Yassin et al. [51]	35 y	M	“Heavy”
Jiménez-Sáenz et al. [52]	31 y	F	>120/HPF
Da Silva et al. [53]	Not specified		Not specified
Amin et al. [54]	37 y	M	“Many”
Karmacharya et al. [55]	45 y	M	“Patchy eosinophilic infiltrate”
Suresh et al. [56]	38 y	M	“Varying numbers”
Atkinson et al. [57]	33 y	F	“Significant”
Inamura et al. [58]	28 y	M	“Numerous”
Ashida et al. [59]	29 y	F	Not quantified
Uchida et al. [60]	48 y	M	“Remarkable”
Katsinelos et al. [61]	72 y	F	“Intense”
Lee et al. [62]	43 y	F	Not quantified
Peršić et al. [63]	8 y	M	“Mainly” eosinophils
Anveden-Herzberg et al. [64]	Infants	5 M, 4 F	>50/HPF
Tajima and Katagiri [65]	44 y	M	Not quantified
Lange et al. [32]	51 y	F	Not quantified
Fraile et al. [66]	38 y	M	Not quantified
Anttila and Valtonen [31]	57 y	M	34/HPF
Bridges et al. [29]	57 y	F	Not quantified
Dunstone [4]	17 y	M	“Considerable numbers”

## References

[1] Yan BM, Shaffer EA (2009). Primary eosinophilic disorders of the gastrointestinal tract. *Gut*.

[2] Alfadda AA, Storr MA, Shaffer EA (2011). Eosinophilic colitis: an update on pathophysiology and treatment. *British Medical Bulletin*.

[3] Kaijser R (1936). Zur Kenntnis der allergischen affektionen des verdauungskanals vom standpunkt des chirurgen aus. *Archiv für Klinische Chirurgie*.

[4] Dunstone GH (1959). A case of eosinophilic colitis. *British Journal of Surgery*.

[5] Dumke K, Janitschke K (1981). Beitrag zur morphologie und pathogenese der eosinophilen kolitis. *Zeitschrift für Gastroenterologie*.

[6] Sherman MP, Cox KL (1982). Neonatal eosinophilic colitis. *Journal of Pediatrics*.

[7] Naylor AR, Pollet JE (1985). Eosinophilic colitis. *Diseases of the Colon and Rectum*.

[8] Moore D, Lichtman S, Lentz J (1986). Eosinophilic gastroenteritis presenting in an adolescent with isolated colonic involvement. *Gut*.

[9] Hill SM, Milla PJ (1990). Colitis caused by food allergy in infants. *Archives of Disease in Childhood*.

[10] Minciu O, Wegmann D, Gebbers JO (1992). Eosinophile Kolitis—eine seltene ursache des akuten abdomens: fallbeschreibungen und literaturubersicht. *Schweizerische Medizinische Wochenschrift*.

[11] Alfadda AA, Storr MA, Shaffer EA (2011). Eosinophilic colitis: epidemiology, clinical features, and current management. *Therapeutic Advances in Gastroenterology*.

[12] Elawad MA, Hill SM, Smith V, Lindley KJ, Milla PJ (2004). Eosinophilic colitis after infancy. *Journal of Pediatric Gastroenterology and Nutrition*.

[13] Behjati S, Zilbauer M, Heuschkel R (2009). Defining eosinophilic colitis in children: insights from a retrospective case series. *Journal of Pediatric Gastroenterology and Nutrition*.

[14] Lowichik A, Weinberg AG (1996). A quantitative evaluation of mucosal eosinophils in the pediatric gastrointestinal tract. *Modern Pathology*.

[15] DeBrosse CW, Case JW, Putnam PE, Collins MH, Rothenberg ME (2006). Quantity and distribution of eosinophils in the gastrointestinal tract of children. *Pediatric and Developmental Pathology*.

[16] Gonsalves N (2007). Food allergies and eosinophilic gastrointestinal illness. *Gastroenterology Clinics of North America*.

[17] Pascal RR, Gramlich TL (1997). Geographic variations of eosinophil concentration in normal colon mucosa. *Modern Pathology*.

[18] Hurrell JM, Genta RM, Melton SD (2011). Histopathologic diagnosis of eosinophilic conditions in the gastrointestinal tract. *Advances in Anatomic Pathology*.

[19] Lucendo AJ (2010). Eosinophilic diseases of the gastrointestinal tract. *Scandinavian Journal of Gastroenterology*.

[20] Odze RD, Bines J, Leichtner AM, Goldman H, Antonioli DA (1993). Allergic proctocolitis in infants: a prospective clinicopathologic biopsy study. *Human Pathology*.

[21] Hwang JB, Moon HP, Yu NK, Sang PK, Suh SI, Kam S (2007). Advanced criteria for clinicopathological diagnosis of food protein-induced proctocolitis. *Journal of Korean Medical Science*.

[22] Lee CK, Kim HJ (2010). Primary eosinophilic colitis as an unusual cause of chronic diarrhea. *Endoscopy*.

[23] Klein NC, Hargrove RL, Sleisenger MH, Jeffries GH (1970). Eosinophilic gastroenteritis. *Medicine*.

[24] Schäppi MG, Smith VV, Milla PJ, Lindley KJ (2003). Eosinophilic myenteric ganglionitis is associated with functional intestinal obstruction. *Gut*.

[25] Cacopardo B (1997). Eosinophilic ileocolitis by Enterobius vermicularis: a description of two rare cases. *Italian Journal of Gastroenterology and Hepatology*.

[26] Al Samman M, Haque S, Long JD (1999). Strongyloidiasis colitis: a case report and review of the literature. *Journal of Clinical Gastroenterology*.

[27] Chandrasekhara V, Arslanlar S, Sreenarasimhaiah J (2007). Whipworm infection resulting in eosinophilic colitis with occult intestinal bleeding. *Gastrointestinal Endoscopy*.

[28] Cuffari C, Oligny L, Seidman EG (1998). Dientamoeba fragilis masquerading as allergic colitis. *Journal of Pediatric Gastroenterology and Nutrition*.

[29] Bridges AJ, Marshall JB, Diaz-Arias AA (1990). Acute eosinophilic colitis and hypersensitivity reaction associated with naproxen therapy. *American Journal of Medicine*.

[30] Saeed SA, Integlia MJ, Pleskow RG (2006). Tacrolimus-associated eosinophilic gastroenterocolitis in pediatric liver transplant recipients: role of potential food allergies in pathogenesis. *Pediatric Transplantation*.

[31] Anttila VJ, Valtonen M (1992). Carbamazepine-induced eosinophilic colitis. *Epilepsia*.

[32] Lange P, Oun H, Fuller S, Turney JH (1994). Eosinophilic colitis due to rifampicin. *The Lancet*.

[33] Iwańczak B, Ruczka M (2011). Eozynofilowe zapalenie jelita grubego w przebiegu nietolerancji preparatów 5-ASA u 8-letniej dziewczynki ze sferocytoza wrodzona. *Pediatr Współcz Gastroenterol Hepatol i Zywienie Dziecka*.

[34] Clouse RE, Alpers DH, Hockenbery DM, DeSchryver-Kecskemeti K (1992). Pericrypt eosinophilic enterocolitis and chronic diarrhea. *Gastroenterology*.

[35] Melton GB, Gaertner WB, MacDonald JE (2011). Eosinophilic colitis: university of Minnesota experience and literature review. *Gastroenterology Research and Practice*.

[36] Yamada Y, Nishi A, Ebara Y (2011). Eosinophilic gastrointestinal disorders in infants: a Japanese case series. *International Archives of Allergy and Immunology*.

[37] Basilious A, Liem J (2011). Nutritional management of Eosinophilic Gastroenteropathies: case series from the community. *Journal of Allergy and Clinical Immunology*.

[38] Durilova M, Stechova K, Petruzelkova L, Stavikova V, Ulmannova T, Nevoral J (2010). Is there any relationship between cytokine spectrum of breast milk and occurence of eosinophilic colitis?. *Acta Paediatrica, International Journal of Paediatrics*.

[39] Piscaglia AC, Larocca LM, Cammarota G (2009). Patchy left-sided colitis: primary eosinophilic colitis or paraneoplastic syndrome?. *Clinical Gastroenterology and Hepatology*.

[40] Chen YJ, Lin YF, Hsieh TY, Chang WK (2010). Eosinophilic colitis. *Digestive and Liver Disease*.

[41] Shim LSE, Eslick GD, Kalantar JS (2008). Gastrointestinal: eosinophilic colitis. *Journal of Gastroenterology and Hepatology*.

[42] Sussman DA, Bejarano PA, Regev A (2008). Eosinophilic cholangiopathy with concurrent eosinophilic colitis in a patient with idiopathic hypereosinophilic syndrome. *European Journal of Gastroenterology and Hepatology*.

[43] Ertuğrul I, Ülker A, Turhan N, Dağli Ü, Şaşmaz N (2008). Eosinophilic colitis as an unusual cause of severe bloody diarrhea. *Turkish Journal of Gastroenterology*.

[44] Velchuru VR, Khan MAB, Hellquist HB, Studley JGN (2007). Eosinophilic colitis. *Journal of Gastrointestinal Surgery*.

[45] Lee JH, Park HY, Choe YH, Lee SK, Il Lee S (2007). The development of eosinophilic colitis after liver transplantation in children. *Pediatric Transplantation*.

[46] Ohtsuka Y, Shimizu T, Shoji H (2007). Neonatal transient eosinophilic colitis causes lower gastrointestinal bleeding in early infancy. *Journal of Pediatric Gastroenterology and Nutrition*.

[47] Lewis JT, Candelora JN, Hogan RB, Briggs FR, Abraham SC (2007). Crystal-storing histiocytosis due to massive accumulation of Charcot-Leyden crystals: a unique association producing colonic polyposis in a 78-year-old woman with eosinophilic colitis. *American Journal of Surgical Pathology*.

[48] Inamura H, Kashiwase Y, Morioka J, Suzuki K, Igarashi Y, Kurosawa M (2006). Accumulation of mast cells in the interstitium of eosinophilic colitis. *Allergologia et Immunopathologia*.

[49] Kandaswamy GV, Kumar ST, Jeyasingh R (2006). Acute abdomen due to eosinophilic colitis with liver abscess. *Indian Journal of Gastroenterology*.

[50] Sierra Salinas C, Blasco Alonso J, Olivares Sánchez L, Barco Gálvez A, Del Río Mapelli L (2006). Allergic colitis in exclusively breast-fed infants. *Anales de Pediatria*.

[51] Yassin MA, Khan FY, Al-Ani A, Fawzy Z, Al-Bozom IA (2005). Ascites and eosinophilic colitis in a young patient. *Saudi Medical Journal*.

[52] Jiménez-Sáenz M, González-Cámpora R, Linares-Santiago E, Herrerías-Gutiérrez JM (2006). Bleeding colonic ulcer and eosinophilic colitis: a rare complication of nonsteroidal anti-inflammatory drugs. *Journal of Clinical Gastroenterology*.

[53] Da Silva JGN, De Brito T, Cintra Damião AOM, Laudanna AA, Sipahi AM (2006). Histologic study of colonic mucosa in patients with chronic diarrhea and normal colonoscopic findings. *Journal of Clinical Gastroenterology*.

[54] Amin MA, Hamid MA, Saba S (2005). Hypereosinophilic syndrome presenting with eosinophilic colitis, enteritis and cystitis. *Chinese Journal of Digestive Diseases*.

[55] Karmacharya R, Mino M, Pirl WF (2005). Clozapine-induced eosinophilic colitis. *American Journal of Psychiatry*.

[56] Suresh E, Doherty V, Schofield O, Goddard C, Dhillon V (2005). Eosinophilic fasciitis and eosinophilic colitis: a rare association. *Rheumatology*.

[57] Atkinson RJ, Dennis G, Cross SS, McAlindon ME, Sharrack B, Sanders DS (2004). Eosinophilic colitis complicating anti-epileptic hypersensitivity syndrome: an indication for colonoscopy?. *Gastrointestinal Endoscopy*.

[58] Inamura H, Tomita M, Okano A, Kurosawa M (2003). Serial blood and urine levels of EDN and ECP in eosinophilic colitis. *Allergy*.

[59] Ashida T, Shimada T, Kawanishi K, Miyatake J, Kanamaru A (2003). Eosinophilic colitis in a patient with acute myeloid leukemia after allogeneic bone marrow transplantation. *International Journal of Hematology*.

[60] Uchida N, Ezaki T, Fukuma H (2003). Concomitant colitis associated with primary sclerosing cholangitis. *Journal of Gastroenterology*.

[61] Katsinelos P, Pilpilidis I, Xiarchos P (2002). Oral administration of ketotifen in a patient with eosinophilic colitis and severe osteoporosis. *American Journal of Gastroenterology*.

[62] Lee JH, Lee JW, Jang CS (2002). Successful cyclophosphamide therapy in recurrent eosinophilic colitis associated with hypereosinophilic syndrome. *Yonsei Medical Journal*.

[63] Peršić M, Štimac T, Štimac D, Kovač D (2001). Eosinophilic colitis: a rare entity. *Journal of Pediatric Gastroenterology and Nutrition*.

[64] Anveden-Hertzberg L, Finkel Y, Sandstedt B, Karpe B (1996). Proctocolitis in exclusively breast-fed infants. *European Journal of Pediatrics*.

[65] Tajima K, Katagiri T (1996). Deposits of eosinophil granule proteins in eosinophilic cholecystitis and eosinophilic colitis associated with hypereosinophilic syndrome. *Digestive Diseases and Sciences*.

[66] Fraile G, Rodriguez-Garcia JL, Beni-Perez R, Redondo C (1994). Localized eosinophilic gastroenteritis with necrotizing granulomas presenting as acute abdomen. *Postgraduate Medical Journal*.

[67] Sunkureddi PR, Luu N, Xiao SY, Tang WW, Baethge BA (2005). Eosinophilic enteritis with systemic lupus erythematosus. *Southern Medical Journal*.

[68] Chen B, Girgis S, El-Matary W (2010). Childhood autism and eosinophilic colitis. *Digestion*.

[69] Uzunismail H, Hatemi I, Doğusoy G, Akin O (2006). Dense eosinophilic infiltration of the mucosa preceding ulcerative colitis and mimicking eosinophilic colitis: report of two cases. *Turkish Journal of Gastroenterology*.

[70] Katsanos KH, Zinovieva E, Lambri E, Tsianos EV (2011). Eosinophilic-Crohn overlap colitis and review of the literature. *Journal of Crohn’s and Colitis*.

[71] Pensabene L, Brundler MA, Bank JM, Di Lorenzo C (2005). Evaluation of mucosal eosinophils in the pediatric colon. *Digestive Diseases and Sciences*.

[72] Choy MY, Walker-Smith JA, Williams CB, MacDonald TT (1990). Activated eosinophils in chronic inflammatory bowel disease. *The Lancet*.

[73] Dehlink E, Fiebiger E (2009). The role of the high-affinity IgE receptor, FcepsilonRI, in eosinophilic gastrointestinal diseases. *Immunology and Allergy Clinics of North America*.

[74] Rothenberg ME (2004). Eosinophilic gastrointestinal disorders (EGID). *Journal of Allergy and Clinical Immunology*.

[75] Okpara N, Aswad B, Baffy G (2009). Eosinophilic colitis. *World Journal of Gastroenterology*.

[76] Hruz P, Straumann A, Bussmann C (2011). Escalating incidence of eosinophilic esophagitis: a 20-year prospective, population-based study in Olten County, Switzerland. *Journal of Allergy and Clinical Immunology*.

[77] Guajardo JR, Plotnick LM, Fende JM, Collins MH, Putnam PE, Rothenberg ME (2002). Eosinophil-associated gastrointestinal disorders: a world-wide-web based registry. *Journal of Pediatrics*.

[78] Yakoot M (2011). Eosinophilic digestive disease (EDD) and allergic bronchial asthma; two diseases or expression of one disease in two systems?. *Italian Journal of Pediatrics*.

[79] Mueller S (2008). Classification of eosinophilic gastrointestinal diseases. *Best Practice and Research*.

[80] Leonard SA, Nowak-Wgrzyn A (2011). Food proteininduced enterocolitis syndrome: an update on natural history and review of management. *Annals of Allergy, Asthma and Immunology*.

[81] Rubin M (1940). Allergic intestinal bleeding in the newborn. *The American Journal of the Medical Sciences*.

[82] Boné J, Claver A, Guallar I (2008). Proctocolitis alérgica, enterocolitis inducida por alimento: mecanismos immunes, diagnóstico y tratamiento. *Allergol Immunopathol*.

[83] Nowak-Wegrzyn A, Sampson HA, Wood RA, Sicherer SH (2003). Food protein-induced enterocolitis syndrome caused by solid food proteins. *Pediatrics*.

[84] Heine RG (2004). Pathophysiology, diagnosis and treatment of food protein-induced gastrointestinal diseases. *Current Opinion in Allergy and Clinical Immunology*.

[85] Sicherer SH, Eigenmann PA, Sampson HA (1998). Clinical features of food protein-induced enterocolitis syndrome. *Journal of Pediatrics*.

[86] Maloney J, Nowak-Wegrzyn A (2007). Educational clinical case series for pediatric allergy and immunology: allergic proctocolitis, food protein-induced enterocolitis syndrome and allergic eosinophilic gastroenteritis with protein-losing gastroenteropathy as manifestations of non-IgE-mediated cow’s milk allergy. *Pediatric Allergy and Immunology*.

[87] Jenkins HR, Pincott JR, Soothill JF (1984). Food allergy: the major cause of infantile colitis. *Archives of Disease in Childhood*.

[88] Sampson HA (1999). Food allergy. Part 1: immunopathogenesis and clinical disorders. *Journal of Allergy and Clinical Immunology*.

